# Emodiversity Among U.S. Emerging Adults: Implications for Health and Wellbeing

**DOI:** 10.3390/bs16010159

**Published:** 2026-01-22

**Authors:** Robert R. Wright, Brandon Jones, Spencer Johnson

**Affiliations:** Psychology Department, Brigham Young University—Idaho, Rexburg, ID 83460, USA; jon21082@byui.edu (B.J.); joh20049@byui.edu (S.J.)

**Keywords:** emodiversity, emerging adults, anxiety, depression, emotions

## Abstract

Emodiversity, or diversity of emotional experience, has received mixed support in the literature as an indicator of health and wellness. The current investigation seeks to contribute to this literature by addressing how the concepts of positive emodiversity and negative emodiversity are related to several wellness indicators (physical, mental, social) within the U.S. emerging adult population (ages 18–30) using cross-sectional and repeated-measures (2 time points) methods. First, in Study 1, positive and negative emodiversity constructs were examined for concurrent relationships with health and wellness indicators among more than 1400 emerging adults. Second, in Study 2, using a Time 1/Time 2 study design, Time 2 health variables were regressed on Time 1 positive and negative emodiversity constructs to examine predictive validity. The results indicated support for positive emodiversity as a concurrent indicator of health and wellness but were not associated with future health and wellness. Negative emodiversity, however, was a poor concurrent indicator but was associated with some indicators of future health and wellness.

## 1. Introduction

As a relatively new construct, emodiversity, or the diversity of emotional experience within the individual, has demonstrated mixed findings for its relative value in the examination of health and wellbeing. While some studies have reported clear benefits to those experiencing greater emodiversity (e.g., Benson et al., 2018; Quoidbach et al., 2014), others have reported inconsistent findings (e.g., Ong et al., 2018; Urban-Wojcik et al., 2022). Despite criticisms of the construct (e.g., Brown & Coyne, 2017) and disparate methodological designs, these results suggest that specific populations, such as emerging adults (ages 18 to 30), may manifest unique associations between emodiversity and health. Indeed, emerging adulthood represents a phase of life wherein emotions play a large role in both future and current health and wellness (Arnett, 2007), making it an important demographic to explore. Yet, few studies have explored emodiversity within this life phase and the emodiversity construct has not been examined using cross-sectional methodology. As such, the current study investigated emodiversity, both positive- and negative-valenced, within two separate studies based on cross-sectional and two-point repeated-measures data gathered among emerging adults in the U.S.

### 1.1. Emodiversity as a Construct Related to Health and Wellness

Emodiversity is based on the concept that a diverse emotional experience should represent a healthy human profile, a more fulfilling or balanced experience and, as such, be related to better health and wellness (Quoidbach et al., 2014). Thus, someone who experiences only happiness would be bereft of the fulness of benefits that come by experiencing a wider range of positive emotions (e.g., relaxed, content, enthusiasm, awe, joy). Moreover, someone who experiences sadness, irritability, boredom, and fear would have a more fulfilling experience than someone who only experiences anger. This diversity of emotional experience is postulated to go beyond simple emotion valence and intensity to provide an additional metric of a fulfilling emotional experience. As such, applied to the individual human experience, similar diversity of emotions is theorized to be indicative of a healthy human profile and, as diversity increases, wellbeing and health should correspondingly improve.

While the connection between diversity in positive affect and wellbeing may be more straightforward, the link between diversity in negative affect and wellbeing seems more nuanced. For instance, as a supporting theoretical perspective, the Broaden and Build Theory (Fredrickson, 2004) postulates that positive emotions, cognitions, and behaviors support and reinforce one another in upward spirals toward increased positivity. Hence, applying this, a diverse array of positive emotions could potentially support each other in positive affectivity and associated wellbeing. However, negative affect is typically associated with poorer wellbeing and long-lasting effects (e.g., negativity bias; Rozin & Royzman, 2001). However, under certain circumstances, an increased diversity of negative emotionality may also be indicative of an individual’s ability to not fixate or ruminate on one particular negative emotion (e.g., anxiety, sadness; D. P. Johnson & Whisman, 2013), which could be associated with improved wellbeing. Thus, in a sense, experiencing a broader range of negative emotions may be indicative of an individual not becoming ensnared or mired in one particular negative emotion.

Originally applied within environmental science, the related concept of biodiversity, typically referred to as Shannon’s entropy, has become a hallmark characteristic of healthy ecosystems (Magurran, 2004; Shannon, 1948). It should be noted that several other approaches to representing diversity exist (see Brown & Coyne, 2017). However, Shannon’s entropy formula allows for a measure of both richness and evenness of diversity rather than one at the expense of the other (Quoidbach et al., 2014). This also allows for a better comparison to studies within the literature that have used this method (e.g., Urban-Wojcik et al., 2022), including the original study by Quoidbach et al. (2014).

In the first attempt to apply this concept, Quoidbach et al. (2014) discovered that a more diverse experience in emotional domains was associated with improved health and wellness. Among two large European sample groups (30,000 and 10,000, respectively), they reported that higher positive emodiversity (PEMO) and negative emodiversity (NEMO) were related to lower levels of depression and fewer doctor visits. Moreover, this relationship remained even after controlling for the influence of positive and negative affect. Subsequent investigations, however, revealed a more complicated relationship for PEMO and NEMO.

### 1.2. Positive Emodiversity and Negative Emodiversity

In relation to PEMO, most studies have found support for a link between higher PEMO and better health and wellness. For instance, using daily diary methodology, Benson et al. (2018) discovered greater self-reported physical health was positively related to PEMO among a sample of midlife American adults. Ong et al. (2018) uncovered a relationship between increased PEMO and lower biomarkers of inflammation in a sample of 688 midlife American adults. Two large studies using ecological momentary assessment through daily diary protocols found additional support. In the first, Urban-Wojcik et al. (2022) examined daily diary data from the Midlife in the U.S. (MIDUS) study with over 2700 midlife Americans. They reported a relationship between PEMO and lower depression, anxiety and physical health symptoms. Second, Lee et al. (2022) used data from MIDUS to examine how emodiversity was related to activity diversity, or engaging in multiple kinds of daily activities. Interestingly, they reported that PEMO was related to greater activity diversity, or engaging in multiple kinds of daily activities. As such, these studies support PEMO as an indicator of health.

NEMO, on the other hand, has been more inconclusive. First, consistent with Quoidbach et al. (2014), Benson et al. (2018) found support for increased NEMO being related to higher self-reported physical health. However, Ong et al. (2018) found no relationship between NEMO and biomarkers of inflammation, suggesting that NEMO was not important, contradicting the findings of Quoidbach et al. (2014) and Benson et al. (2018). In support of NEMO possibly not being a marker of health, Werner-Seidler et al. (2020) reported higher rates of NEMO among a small sample of adult Americans who were clinically depressed. Providing further mixed findings, NEMO was related to higher depression, anxiety, and physical health symptoms among midlife Americans in Urban-Wojcik et al. (2022), whereas Lee et al. (2022) reported that NEMO was related to greater activity diversity.

Taken together, these findings are inconsistent and contradictory. While some studies suggest that NEMO and PEMO are associated with improved health, other studies point to a more complicated interpretation, such as that NEMO may be related to poorer health outcomes under certain conditions. While it is difficult to clearly ascertain the relative impact on these disparate findings, methodological differences (e.g., emodiversity computation), sample characteristic (e.g., clinical vs. population), and developmental stage (e.g., age) in previous studies likely all contribute. One outstanding characteristic of these studies, for instance, is the predominant focus on midlife and older adult populations, rather than on emerging adults.

### 1.3. Emodiversity Among the Emerging Adult Population

Emerging adulthood (ages 18–30) is a phase of life wherein attitudes have not yet crystallized, emotions play a large role in daily living, self-concept is still forming, and they are more susceptible to social influence (Arnett, 2007; Coccia & Darling, 2016; Sears, 1986). All these characteristics of emerging adulthood suggest that emodiversity may likely play an important role, even a disproportionately higher role than in other life stages, within their overall health and wellness. However, only a select few studies have been conducted among emerging adults regarding emodiversity. For instance, Yoon and Kim (2024) examined a sample with an age range of 24–70 years in South Korea, which included emerging adults. They reported that increased PEMO was associated with increased wellbeing, but not NEMO, again suggesting that NEMO may not always be a marker of health and wellness. In a different investigation, Heshmati et al. (2023) explored emodiversity within a Hispanic adolescent population (ages 14–17), which was not an emerging adult population but was younger than previous studies. Using daily diary methodology, they identified that higher rates of NEMO were related to emotional eating. However, while NEMO was linked to maladaptive behavior, PEMO was not, suggesting that under certain conditions, PEMO may not be associated with health benefits either.

In one of two studies exclusively focused on emerging adults, Forster and Lougheed (2025) examined emodiversity within an emerging adult population (ages 17–30) of undergraduate students in western Canada using daily diary methodology during the COVID-19 pandemic restrictions. Whereas low NEMO was associated with higher depressive symptoms and anxiety, as well as lower wellbeing, PEMO was not associated with any of these health metrics. Collectively, these studies conducted among emerging adults provide further contradicting evidence regarding emodiversity and health. It is important to note that these studies had unique characteristics beyond age that may have influenced results (e.g., outside the U.S., COVID-19 pandemic). As such, limited insight regarding how emodiversity may operate among emerging adults in the U.S. regarding health and wellness is currently available. Moreover, even among these few studies, there are inconsistencies which warrant further investigation. Although the investigation of emodiversity among emerging adults in the U.S. will likely not resolve the contradictory findings in the extant literature, it will address the gap in the literature regarding how emodiversity is related to health and wellness within this important developmental stage of life. Studies have highlighted the need to further refine the concept and application of emodiversity (Brown & Coyne, 2017; L. Johnson et al., 2025; Urban-Wojcik et al., 2022), and the U.S. emerging adulthood population represents an important area for such investigation.

### 1.4. The Present Investigation

The present investigation examined the concept of positive and negative emodiversity within a sample of U.S. emerging adults ages 18 to 30 using cross-sectional and two-point repeated measures data within two studies. Given the inconsistencies across the entire literature and among the few studies examining emerging adults, we proposed two non-directional research questions, one for positive emodiversity (PEMO) and one for negative emodiversity (NEMO). Moreover, we included a variety of health and wellness constructs to enhance the ability to investigate these relationships and provide further confidence in the results across three domains (physical, mental, and social). Specifically, we examined subjective physical health and physical symptoms to represent physical health; satisfaction with life, body appreciation, depressive symptoms, perceived stress, and anxiety to represent mental health; and social integration, loneliness, and interpersonal conflict to conceptualize social health. We selected a broad range of wellbeing variables with each of them based on previous studies that have demonstrated links between these health and wellness variables with measures of emotionality (e.g., Quoidbach et al., 2014; Wright et al., 2017, 2023a, 2023b).

RQ1: What is the relationship of positive emodiversity (PEMO) to physical health (subjective physical health and physical symptoms), mental health (anxiety, depressive symptoms, perceived stress, satisfaction with life, and body appreciation), and social health (interpersonal conflict, loneliness, and social integration) indicators among an emerging adult population?

RQ2: What is the relationship of negative emodiveristy (NEMO) to physical health (subjective physical health, and physical symptoms), mental health (anxiety, depressive symptoms, perceived stress, satisfaction with life, and body appreciation), and social health (interpersonal conflict, loneliness, and social integration) indicators among an emerging adult population?

We examined these research questions in two studies. First, we calculated emodiversity based on cross-sectional retrospective data regarding emotional experience over the past 30 days and examined how PEMO and NEMO were related to health. Second, we calculated PEMO and NEMO similarly in a Time 1/Time 2 study design so that Time 1 estimates could be used to predict health outcomes at the Time 2 measurement.

## 2. Study 1

### 2.1. Method

#### Participants and Procedure

In accordance with the Declaration of Helsinki, approval from the local institutional ethics board at Brigham Young University—Idaho was obtained on 17 May 2022 (#S22-02). Prospective student participants within introductory psychology courses on campus were solicited by email invitation. Students were required to participate in research for course credit and allowed to select from several options, including the current study. Students followed a link wherein they provided consent and completed an online questionnaire pertaining to college student life experiences at a location of their choice. Informed consent for participation was obtained from all subjects involved in this study, and data were collected across multiple semesters using this same procedure spanning the years 2023 to 2024. Completion of the online questionnaire took a median time of 53.55 min. After identifying responses that indicated an age below 18 or above 30, a failure on the attention check (indicating they had not completed the survey to the best of their ability), and a lack of permission for publication purposes, 63 responses were removed. Participants (*n* = 1411) were an average of 20.24 (*SD* = 2.09) years of age (range between 18–30) and comprised of mostly women (60.2%). Moreover, most of the sample indicated White ethnicity (85.5%) with Hispanic/Latino(a) (5.7%), Black (2.3%), Asian (1.6%), Native American (0.5%), Native Hawaiian (0.6%), and Other/More than one (3.8%) represented. Most participants were Freshmen (58.0%) and Sophomore (28.8%), with 47.7% of the entire sample indicating they were first semester students. Relationship status was single for most (63.1%), though being in a committed relationship (24.8%), engaged to be married (4.5%), married (7.4%), and divorced/separated (0.2%) were also observed. On average, student participants were taking 12.75 (*SD* = 2.17) credits and, while about half were not currently employed (54.6%), many indicated having a part-time job (41.9%). Participants came from families of relatively high socioeconomic status, where the average education level was 3.48 (*SD* = 1.27; 3 = mother/father received a bachelor’s degree, 4 = mother/father received a master’s degree), and the average income was 4.40 (*SD* = 1.40; 4 = USD 75,000–USD 100,000, 5 = USD 100,000–USD 150,000).

### 2.2. Measures

#### 2.2.1. Demographic Constructs

The questionnaire queried a range of demographic information, including age, gender, relationship status, ethnicity, education level, credit enrollment, and employment status. In addition, socioeconomic status (SES) was assessed using two questions (Wright et al., 2025). One regarding parental education with six categories of increasing education (1 = both mother and father have no college education, 6 = mother or father received advanced training, e.g., medical school, law school) and the other focused on household income for the past 12 months on a seven-point scale (1 = <USD 25,000, 7 = >USD 150,000). For both SES questions, “decline to respond” and “do not know” options were provided but were not included in analyses.

#### 2.2.2. Emotional Experience

Differential Emotion Scale. Positive and negative emotional experiences were examined using the Differential Emotion Scale (Philippot et al., 2003). This measure captured nine positive (e.g., awe, joy, hope) and nine negative emotional states (e.g., fear, anxiety, shame) on a five-point frequency scale (1 = never, 5 = most of the time) during the past month. Both demonstrated acceptable internal consistency (*α*’s = 0.82, 0.83, respectively).

Positive and Negative Emodiversity. Closely following the computational formula provided by Shannon’s entropy and mirroring Quoidbach et al. (2014) and others (e.g., Urban-Wojcik et al., 2022), we calculated both positive and negative emodiversity. Using the positive and negative emotions within the Differential Emotion Scale, we computed positive and negative emodiversity indexes. Specifically, we divided the frequency of one emotional experience by the total number of frequencies of all types of emotion, which provides a proportion of that emotion within the larger emotional valence (i.e., positive, negative). Then, we multiplied this proportion by its natural log, which produced a transformed value for computation. Next, we repeated this for each emotion represented, so that each emotion had a transformed value. Finally, we summed all these values to represent emodiversity within positive and negative domains, respectively and multiplied each total by −1. This produces a scale where higher values represent a more diverse emotional experience.

#### 2.2.3. Physical Health

Subjective Overall Health. Subjective physical health was measured using the single-item EuroQol Fifth Dimension (Kind et al., 2005), where participants rated their own physical health on a scale from 0 (worst physical health) to 100 (best physical health).

Physical Symptom Inventory. Physical health symptoms were measured using Spector and Jex’s (1998) 18-item Physical Symptom Inventory (e.g., headache, fatigue). Participants indicated the presence of any of these symptoms during the past 30 days. As a checklist, no internal consistency estimates were calculated.

#### 2.2.4. Mental Health

Satisfaction with Life Scale. Using a seven-point scale, satisfaction with life (Diener et al., 1985) was captured with five items where participants indicated their level of agreement regarding their current life appraisal (1 = strongly disagree, 7 = strongly agree). One item reads, “In most ways, my life is close to my ideal”. Internal consistency was acceptable (*α* = 0.87).

Body Appreciation Scale. Body appreciation (i.e., body image) was examined on a seven-point agreement scale (1 = not at all true, 7 = very true) with the thirteen-item Body Appreciation Scale (Avalos et al., 2005). Higher scores represent greater appreciation for one’s physical body. A representative item includes “I feel good about my body.” Internal consistency was acceptable (*α* = 0.94).

CESD-5. Acute depressive symptomology during the past week was captured using the CES-D five-item measure (Bohannon et al., 2003) on a four-point scale (1 = rarely or none of the time; 4 = most or all of the time). A sample item was “I felt depressed.” Scale internal consistency was acceptable (*α* = 0.75).

Perceived Stress Scale. Using Cohen et al.’s (1983) Perceived Stress Scale, overall life stress was examined using seven items on a five-point frequency scale (1 = never, 5 = very often). One of the questions in this scale was “how often have you felt that you were unable to control the important things in your life?” Internal consistency was acceptable for this scale (*α* = 0.81).

Anxiety Scale. Using the same five-point frequency scale as above, anxiety during the past three months was assessed using the four-item measure (anxious, worried, at ease, and comfortable) from Butz and Yogeeswaran (2011). Internal consistency was acceptable (*α* = 0.82).

#### 2.2.5. Social Health

Social Integration Scale. The eight-item measure of social integration (i.e., in-person social interactions) on a daily frequency scale during the past month by Twenge et al. (2017) was used to capture behavioral social support. Sample behaviors include going shopping, going to parties or other social activities, and getting together with friends informally. As a behavior checklist, internal consistency estimates were not computed.

Short Loneliness Scale. Using the three-item Short Loneliness Scale (Hughes et al., 2004), perceived loneliness during the past month was assessed on a five-point frequency scale (1 = never, 5 = all the time). A sample item reads, “How often do you feel left out?” Acceptable internal consistency was observed (*α* = 0.84).

Workplace Interpersonal Conflict Scale. Using a modified version of the six-item measure from Wright et al. (2017), interpersonal conflict with others in general (not in the workplace) was assessed on a five-point frequency scale (1 = never, 5 = very often) for the past 3 months. An example item reads, “how often have you felt like you were treated unfairly by others?” The internal consistency of the measure was acceptable (*α* = 0.87).

### 2.3. Data Analysis

First, to determine relationships between the emodiversity constructs and health variables, we computed correlations. Next, to control for the influence of positive and negative affect, we conducted a series of regression analyses where each outcome variable was regressed on the mean affect variable and the corresponding emodiversity variable. This enabled the identification of any unique relationship between the health variable and emodiversity above and beyond the relationship between the health variable and affect. We also included gender (recoded so 0 = male, 1 = female) and age (mean-centered) as control variables in these models. Following generally accepted statistical protocol (Howell, 2020), we computed regression analyses only for those outcome variables demonstrating a significant bivariate correlation with the emodiversity variable. To protect against possible Type I error, which may be inflated by conducting multiple significance tests, we applied Bonferroni’s correction in determining statistical significance.

### 2.4. Results

Means, standard deviations, and other descriptive information are provided in Table 1.

PEMO and NEMO were significantly positively related (*r* = 0.25, *p* < 0.001). Positive affect was related to PEMO (*r* = 0.48, *p* < 0.001), and negative affect was related to NEMO (*r* = 0.19, *p* < 0.001). The participants reported experiencing significantly higher positive affect (*M* = 3.49, *SD* = 0.63) than negative affect (*M* = 2.24, *SD* = 0.64; *t*[1410] = 46.65, *p* < 0.001, *d* = 2.48). Moreover, the participants experienced significantly higher PEMO (*M* = 2.16, *SD* = 0.03) than NEMO (*M* = 2.12, *SD* = 0.04; *t*[1410] = 30.45, *p* < 0.001, *d* = 1.62). These findings suggest that the participants were experiencing nontrivial higher levels of positive affect and PEMO than their respective counterparts (negative affect, NEMO).

#### 2.4.1. Research Question 1 (Positive Emodiversity)

In accordance with our first research question, we examined correlations between positive emodiversity (PEMO) and each of our health outcomes. Eight of the ten physical, mental, and social health variables were significantly related to PEMO (see Table 1), such that increased PEMO was associated with better health. The two exceptions were the number of physical symptoms and interpersonal conflict.

Next, we examined each of the statistically significant bivariate correlation relationships in individual linear regression models that included positive affect, gender, and age as control variables to account for shared variance (see Table 2). Overall, the pattern of results suggested that physical health was not explained by PEMO beyond that which affect can already explain, as subjective physical health was not related to PEMO when positive affect was included. Mental and social health, however, were different. For three of the mental and social health outcomes (perceived stress, anxiety, loneliness), PEMO accounted for variation beyond positive affect. However, exceptions included life satisfaction, depressive symptoms, body appreciation, and social integration.

It should also be noted that in many of the models, the direction of the relationship between PEMO and the health outcome variable changed when positive affect was included as a control variable. This is likely due to multicollinearity between these two variables such that the negative bivariate relationship between PEMO and the outcomes is largely driven by shared variance with positive emotion (*r* = 0.48). In adjusting for positive affect, the PEMO coefficient reflects the association of residual PEMO (that is, PEMO beyond what would be expected given an individual’s level of positive affect), which in these results is positively associated with perceived stress, anxiety, and loneliness, consistent with a suppression effect. This implies that among individuals who exhibit unusually high levels of PEMO relative to their mean positive affect (a subset that is likely small given the strength of the PEMO-positive affect correlation), perceived stress, anxiety, and loneliness outcomes are worse rather than better. Thus, in response to RQ1, we found PEMO to be a significant predictor beyond that accounted for by positive affect, gender, and age for three of the eight variables statistically related to PEMO.

#### 2.4.2. Research Question 2 (Negative Emodiversity)

Using a similar analytic strategy for our second research question regarding negative emodiversity (NEMO), we discovered few associations. In fact, only two of the health variables were related to NEMO: anxiety and interpersonal conflict. Anxiety had a negative relationship, such that as NEMO was higher, anxiety was lower. However, interpersonal conflict was in the opposite direction, suggesting that NEMO was associated with higher conflict. These findings contradict each other, suggesting that NEMO may exhibit differential associations with different facets of health and wellbeing.

As there were two health variables that produced statistically significant correlations with NEMO, only two linear regressions were conducted (see Table 3). NEMO remained a significant factor in explaining anxiety and interpersonal conflict beyond negative affect in the regression models, respectively. However, these were in opposing directions, suggesting that NEMO may have further nuanced or even conflicting associations with concurrent health and wellness, despite controlling for negative affect, gender, and age.

### 2.5. Discussion

The findings from Study 1 suggest that PEMO is associated with healthier profiles. Specifically, these results suggest that higher PEMO corresponds with better subjective health, body appreciation, perceived stress, anxiety, loneliness, and social integration. Moreover, these relationships were robust, such that PEMO accounted for variation in these health variables beyond positive affect, gender, and age. This is in line with the literature that has reported that increased PEMO is related to better health profiles (Quoidbach et al., 2014; Ong et al., 2018; Urban-Wojcik et al., 2022; Yoon & Kim, 2024) and is consistent with theories regarding connections between positive emotions, cognitions, and behaviors (Fredrickson, 2004).

The results provide limited support for NEMO as a variable related to health and the few findings that did were in opposing directions. As such, these results provide further contradicting evidence where NEMO has emerged as a positive predictor of health in some (Benson et al., 2018; Quoidbach et al., 2014), negative in some (Forster & Lougheed, 2025; Urban-Wojcik et al., 2022; Werner-Seidler et al., 2020), and a non-predictor in others (Ong et al., 2018; L. Johnson et al., 2025). While anxiety seems lower when NEMO is higher, interpersonal conflict seems to be the opposite, which suggests that NEMO does not have a unilateral positive or negative influence on overall health across mental and social domains. Offering some potential explanation, interpersonal conflict is associated with a greater range of negative emotions (Wright et al., 2017) and, of all the negative emotions queried, anxiety was the most frequently reported. Thus, this suggests a potential dampening influence of anxiety on NEMO, such that when anxiousness was higher, other negative emotions were lower.

## 3. Study 2

Building on the limitation of the one-time cross-sectional design in Study 1, we conducted Study 2. Specifically, data based on retrospective reports of affect were used to compute PEMO and NEMO in a Time 1/Time 2 study design to then predict subsequent health outcomes three months later, at the Time 2 assessment.

### 3.1. Method

#### Participants and Procedure

In accordance with the Declaration of Helsinki, following ethics board approval at Brigham Young University—Idaho on 3 April 2024 (#W24-23), participants were solicited among students within two upper division psychology courses by email invitation. Students were required to participate for course credit but were given the option to complete an alternative assignment. Prospective participants followed a link and completed an online questionnaire pertaining to their experience in the course to evaluate course effectiveness in a location of their choice. Informed consent for participation was obtained from all subjects involved in this study, and data were collected across multiple semesters spanning the years 2024 to 2025. Completion of the online questionnaire took a median of 33.68 min. Of the total 288 responses, 14 were removed due to age being above 30, failure on the attention check, or because they did not provide permission to use their data for publication purposes. A total of 47 completed the first questionnaire and did not complete the second (17% attrition). Participants (*n* = 274) were an average of 22.73 (*SD* = 2.35) years of age (range of 18–30 years) and comprised of slightly more women (55.1%). Moreover, most of the sample indicated White ethnicity (82.8%) with Hispanic/Latino(a) (9.5%), Black (1.8%), Asian (1.1%), Native Hawaiian (1.1%), and Other/More than one (3.6%) represented. Most participants were Seniors (44.2%) and Juniors (37.2%). Relationship status of single was most common (43.1%), though married (36.1%), being in a committed relationship (16.8%), engaged to be married (2.6%), and divorced/separated (1.5%) were also observed. On average, student participants were taking 12.68 (*SD* = 2.67) credits, and most were currently employed (63.1%). Participants came from families of relatively high socioeconomic status, where the average education level was 3.48 (*SD* = 1.30; 3 = mother/father received a bachelor’s degree, 4 = mother/father received s master’s degree) and the average income was 4.20 (*SD* = 1.48; 4 = USD 75,000–USD 100,000, 5 = USD 100,000–USD 150,000).

### 3.2. Measures

The measures in Study 2 were identical to those in Study 1. Internal consistency estimates for the Differential Emotion Scale (Philippot et al., 2003) were acceptable at both time points for positive (*α*’s = 0.80, 0.83, respectively) and negative affect (*α*’s = 0.80, 0.81, respectively). All other internal consistency estimates are reported in Table 4.

### 3.3. Data Analysis

We followed a similar analytic strategy to that of Study 1. To determine the relationships between the emodiversity constructs at Time 1 and health variables at Time 2, we computed correlations. Next, to control for the influence of positive and negative emotion, we conducted a series of regression analyses where each outcome variable at Time 2 was regressed on the emotion variable at Time 1 and the corresponding emodiversity variable at Time 1. We also included the wellness outcome variable at Time 1 to help establish directionality of relationships. We computed these regression analyses for those outcome variables at Time 2 that demonstrated a significant relationship with the emodiversity variable at Time 1 and applied Bonferroni’s correction when determining statistical significance. Moreover, we included gender (recoded so 0 = male, 1 = female) and age (mean-centered) as control variables in these analyses. Finally, we explored reverse directionality by modeling health outcome variables at Time 1 as predictors for the emodiversity variables at Time 2 for each original regression model conducted.

### 3.4. Results

Means, standard deviations, and other descriptive information are provided in Table 4. PEMO and NEMO were significantly and positively correlated at Time 1 (*r* = 0.13) and at Time 2 (*r* = 0.21). Suggesting possible trait-like tendencies, PEMO at Time 1 was correlated to PEMO at Time 2 (*r* = 0.46), and NEMO showed a similar pattern (*r* = 0.50). Participants experienced similar levels of PEMO (*t*[508] = 0.79, *p* = 0.428) and NEMO (*t*[508] = 1.52, *p* = 0.129) at both time points. Age, SES education, and SES income were not significantly related to PEMO or NEMO at either time point, with one exception: SES income was positively related to PEMO at Time 1 (see Table 4). PEMO did not differ between the genders. However, women reported significantly less NEMO than men at Time 1 (*M_Women_* = 2.11, *SD* = 0.05; *M_men_* = 2.13, *SD* = 0.04; *t*[266] = 3.29, *p* = 0.001) and at Time 2 (*M_Women_* = 2.11, *SD* = 0.05; *M_men_* = 2.13, *SD* = 0.04; *t*[234] = 3.40, *p* < 0.001).

#### 3.4.1. Research Question 1 (Positive Emodiversity)

Correlation analyses revealed that PEMO at Time 1 was significantly related to six wellness variables at Time 2 (depressive symptoms, satisfaction with life, body appreciation, perceived stress, anxiety, and loneliness). Therefore, six separate multivariate regression analyses were conducted where these six Time 2 variables were individually regressed on PEMO at Time 1 controlling for the respective wellness variable at Time 1, positive affect at Time 1 as well as gender and age (see Table 5). All six regression analyses revealed nonsignificant (*p*’s > 0.05) relationships. As such, PEMO was not associated with future health and wellness beyond positive affect.

#### 3.4.2. Research Question 2 (Negative Emodiversity)

Following the same analytic procedure, NEMO at Time 1 was significantly related to three wellness variables at Time 2 (depressive symptoms, anxiety, and life satisfaction). Three separate multivariate regression analyses were conducted, such that each of these three Time 2 variables were regressed on NEMO at Time 1, controlling for the respective wellness variable at Time 1, negative affect at Time 1, as well as gender and age (see Table 6). Each of these analyses resulted in statistically significant relationships, suggesting that NEMO was associated with each of these wellness variables beyond that explained by negative affectivity and Time 1 levels. Specifically, NEMO was positively related with life satisfaction, as well as negatively associated with both depressive symptoms and anxiety, suggesting that higher NEMO was associated with future wellness improvements.

#### 3.4.3. Alternative Explanations Analyses

For each of the six PEMO and three NEMO regression analyses conducted for RQ1 and RQ2, we conducted reverse directionality analyses, such that Time 2 PEMO and Time 2 NEMO, respectively, were regressed on the outcome variables at Time 1 (see Table 7). Notably, nearly every bivariate relationship that was statistically significant in the original analyses was also statistically significant in these analyses. While these results suggest potential for reverse directionality, it is important to note that across all nine of these analyses, *R*^2^ was substantially greater for the original direction (emodiversity predicting health outcomes) compared to the reverse without the controls for gender and age (*M* = +0.10; range: +0.05 to +0.19). Thus, the greatest explanatory power was observed in the original direction.

### 3.5. Discussion

The findings from Study 2 suggest that PEMO at Time 1 was not associated with improved health outcomes at Time 2. This suggests that PEMO is not predictive of health outcomes for emerging adults or, at least not for the variables considered as part of mental, physical, and social wellness in the current investigation. PEMO may be a construct that is more sensitive to, or prone to change based on, a person’s circumstances (e.g., daily uplifts), which may be perceived differently over time (Park et al., 2004). However, our analyses uncovered a moderate correlation of PEMO at Time 1 and 2, suggesting some consistency in experiencing PEMO within person. Alternatively, it is possible that methodological characteristics (e.g., PEMO construct creation from retrospective data), sample demographics (e.g., emerging adulthood), or historical characteristics (e.g., time within semester) influenced these results.

Interestingly, NEMO at Time 1 was associated with improvements in three of the health outcomes at Time 2 while controlling for Time 1 levels of each respective health outcome and negative affect. This lends further support to the notion that having a diverse negative emotional experience is indicative of better health and wellness (Forster & Lougheed, 2025; Quoidbach et al., 2014). NEMO may be particularly important as it may provide protection from an individual delving too deeply into any one negative emotion (e.g., anxiety, sadness) and, thereby, prevent poor mental health such as depression and anxiety. This may also extend to improvements in life satisfaction. However, NEMO may not impact other health and wellness variables, as our results suggest the other seven variables were not related to NEMO. Similar to PEMO, a moderate correlation of NEMO at Time 1 and 2 was observed, suggesting consistency in experiencing NEMO over time.

## 4. General Discussion

The current investigation comprised two studies aimed at examining the constructs of positive and negative emodiversity and their associations with health and wellness variables among an U.S. emerging adult population using cross-sectional data. Study 1 results demonstrated several significant concurrent associations between positive emodiversity (PEMO) and better health while uncovering only a few health associations for negative emodiversity (NEMO). Study 2, on the other hand, established some predictive validity of NEMO for the three wellness outcomes of satisfaction with life, depressive symptoms, and anxiety, whereas PEMO demonstrated no such relationship. Collectively, these results support emodiversity as a health and wellness construct among emerging adults and suggests that cross-sectional assessments of emotional experience may be used to examine emodiversity and related health and wellness, including future health outcomes. These findings have important implications for understanding emodiversity and future research in affect and wellness, particularly among emerging adults.

### 4.1. PEMO and Health Among Emerging Adults

First, consistent with prior research (Benson et al., 2018; Ong et al., 2018; Quoidbach et al., 2014; Urban-Wojcik et al., 2022; Yoon & Kim, 2024) and theory, including the broaden and build theoretical framework (Fredrickson, 2004), the findings suggest that PEMO is modestly related to many wellness variables across physical, mental, and social domains for emerging adults. Indeed, these findings support a potential mechanism wherein a more diverse experience of positive emotions may, at least in the short term, perpetuate a cycle of positive emotions, cognitions (e.g., positive affirmations), behaviors (e.g., daily uplifts), and health in an upward, interdependent spiral. This seems to be particularly true for concurrent associations such that when PEMO is higher, health and wellbeing is higher and these likely are interdependent in lifting and supporting each other.

The observation that PEMO is concurrently but not predictively associated with increased health and wellness beyond the influence of positive affect suggests a more nuanced relationship over time, however, and is contrary to other findings in the literature regarding the main effects of positive affect (e.g., Catalino & Tov, 2022). Importantly, this association refers to diversity in positive emotion, not to experiencing positive emotion itself. Indeed, it may be that PEMO is more contingent on occurrences of events that may not be in one’s control (i.e., interpersonal daily uplifts), particularly during emerging adulthood (Arnett, 2007). Moreover, the relationship between PEMO and wellbeing has been identified in studies that used ecological momentary assessment, which is adept at capturing associations within a closer, momentary timeline (Benson et al., 2018; Quoidbach et al., 2014; Urban-Wojcik et al., 2022). However, the three-month interval between time points in Study 2 may have been long enough to dissociate PEMO from subsequent wellbeing. Taken together, consistent with diversity theory and the literature (Magurran, 2004; Quoidbach et al., 2014), as PEMO increases, better health profiles coincide but may not persist in the long term.

Building on this idea, PEMO is likely further influenced by individual perceptions and appraisals, which are profoundly impacted by other exogenous (e.g., stressors, demands; Lazarus & Folkman, 1984) and endogenous factors (e.g., happiness valuation, optimism; Mauss et al., 2011), which may be particularly potent during emerging adulthood (Arnett, 2007). After a period, the beneficial influence of PEMO, if not sustained by other cognitions and behaviors (Fredrickson, 2004), may lose their strength in promoting health. Indeed, as suggested by the Evaluative Space Model (Cacioppo & Berntson, 1994), baseline emotionality may be positively skewed (positivity offset), such that positive emotions may be assumed as the norm, requiring substantial and sustained positive input to effect long-term changes. As such, PEMO may not be a durable predictor of future health outcomes for emerging adults, but rather a momentary indicator of a more fulfilling life, particularly in the U.S.

### 4.2. NEMO and Health Among Emerging Adults

Second, the opposing pattern of results for NEMO offers insight into a complicated process that is both consistent with some of the literature and with theories on negativity bias (Rozin & Royzman, 2001) and rumination (D. P. Johnson & Whisman, 2013). For instance, in Study 1, few wellness variables were related to NEMO, and these were inconsistent in direction, suggesting NEMO may not have a unilateral healthy influence for emerging adults, which has been suggested among midlife and older adults (e.g., Ong et al., 2018; Urban-Wojcik et al., 2022; Werner-Seidler et al., 2020). However, the Study 2 findings provided a contrast, as NEMO demonstrated a relationship with future satisfaction with life, depressive symptoms, and anxiety, consistent with previous studies of NEMO having a positive influence on health (Benson et al., 2018; Forster & Lougheed, 2025; Quoidbach et al., 2014). This finding supports the presence of a negativity bias, such that the influence of negative emotions and cognitions on overall wellness may supersede the positive (Rozin & Royzman, 2001) and have longer duration (Larsen, 2009), albeit in a positive manner. This may be counterintuitive, but it is important to note that negative affect is typically associated with poorer wellbeing and long-lasting effects (e.g., negativity bias; Rozin & Royzman, 2001), but NEMO represents diversity and balance of negative emotionality.

Indeed, it may be that more balance in negative affectivity exerts a lagged, though robust, influence beyond what the more transient balance of positive emotions (PEMO) can provide. Rumination theory provides some insight into this potential mechanism. Even as an individual dwells on specific negative thoughts and emotions to the point of fixation, this may inhibit the ability to experience other emotions (D. P. Johnson & Whisman, 2013). This may be applicable to those with depressive symptoms or high levels of anxiety, where the individual may focus on specific negative emotions (e.g., sadness, worry) at the expense of other emotions. Thus, these findings may suggest that an increased diversity of negative emotionality may be indicative of an individual’s ability to not fixate or ruminate on one particular negative emotion, which could be associated with improved wellbeing. In the short term, therefore, it may be that NEMO is inconsistent, but over a period of time, may offer counteracting effects to protect against detrimental wellness outcomes, particularly among emerging adults, for whom many experiences are novel and emotionally stimulating (Coccia & Darling, 2016).

### 4.3. Potential Limitations and Future Research

Notwithstanding the contributions of this investigation, potential limitations should be acknowledged. First, our data were cross-sectional, precluding strong causal inferences or clear interpretations of momentary processes. Even the repeated-measures design in Study 2 was limited with only two assessments. However, our use of two study designs bolsters confidence in the results. Second, our methodological design may present additional challenges. For instance, the interpretation of the multivariate regression models was hampered by the inherent multicollinearity in the model, as emodiversity was calculated from the same variables as affect, with an applied transformation. Moreover, the self-report and temporal nature of the data introduce potential subjective and retrospective biases that cannot be controlled or parceled out. Nevertheless, emotional experience is subjective, making this type of bias unavoidable to some extent. Third, although we selected constructs from previous studies, the health and wellness constructs in this investigation may not offer a comprehensive view of health and wellness, as the constructs selected were not equal in number across the domains (physical, mental, and social), and other constructs may have been omitted. Finally, our college student sample and cohort characteristics may hamper generalizability for other groups, even emerging adults, such as those not attending higher education, differing ethnicity, low socioeconomic status, or different cultural perspectives.

Future research should seek to expand upon these limitations and build on the findings from this study. As this work was exploratory in nature, further work should be done among emerging adults in both college and other contexts to confirm these findings using both cross-sectional and ecological momentary assessment designs for both concurrent and predictive validity. The predominant approach to examining emodiversity has been to use ecological momentary assessment, which provides insight into the unique ebb and flow of phenomena (Park et al., 2004). Although typically regarded as a superior approach to studying fluctuating and dynamic processes, ecological momentary assessment can be expensive, time-intensive, and difficult to conduct (Shiffman et al., 2008). Despite the methodological challenges (e.g., retrospective bias), cross-sectional data seem appropriate to use in building estimates of emodiversity. As such, future studies should consider using cross-sectional and short repeated measures designs, such as employed here, to further explore emodiversity influences and correlates that may transcend the momentary experience.

Building on our results of possible stability over time in the diverse emotional experience, future research could explore how PEMO and NEMO may be related to dispositional characteristics, which may be helpful in emerging adult theory. Future examinations should also consider additional variables (e.g., grades, work performance) and more objective biometrics (e.g., blood pressure). Finally, influential factors for emerging adults such as gender, socioeconomic status, technology use, and romantic relationships (Wright et al., 2024) should be further investigated relative to emodiversity.

## 5. Conclusions

In conclusion, our use of two study designs in the current investigation to establish both concurrent and predictive validity of positive and negative emodiversity constructs among an emerging adult population provides a meaningful contribution to the literature. Offering insight into the operation of complex affective processes that influence wellbeing, the results uncovered an association of diverse emotional experience within positive and negative domains and both concurrent and future health and wellness. Moreover, this investigation provides evidence that emodiversity constructs can be effectively applied in examining health and wellness by using cross-sectional data. Going forward, emodiversity could be considered in many other contexts and methodological designs as an indicator of health and wellness.

## Figures and Tables

**Table 1:** Study 1: Correlations between emotional and health variables.

Variable	*M* (*SD*)	PA	NA	PEMO	NEMO
**Demographics**					
Age	20.24 (2.09)	0.04	−0.08 ***	0.03	0.03
SES Education	3.48 (1.27)	−0.01	−0.07 ***	0.02	−0.03
SES Income	4.40 (1.40)	−0.01	−0.10 ***	0.02	−0.01
**Physical Health**					
Subjective Health	79.65 (13.56)	0.27 ***	−0.27 ***	0.13 ***	−0.01
Physical Symptoms	5.45 (3.59)	−0.12 ***	0.40 ***	−0.06	−0.02
**Mental Health**					
Depressive Symptoms	8.71 (3.13)	−0.41 ***	0.61 ***	−0.15 ***	−0.06
Satisfaction with Life	4.70 (1.28)	0.59 ***	−0.38 ***	0.23 ***	0.03
Body Appreciation	5.12 (1.27)	0.34 ***	−0.42 ***	0.15 ***	0.02
Perceived Stress	2.66 (0.64)	−0.50 ***	0.59 ***	−0.14 ***	−0.05
Anxiety	2.84 (0.75)	−0.46 ***	0.66 ***	−0.13 ***	−0.16 ***
**Social Health**					
Loneliness	2.67 (0.92)	−0.40 ***	0.45 ***	−0.12 ***	−0.02
Interpersonal Conflict	1.91 (0.69)	−0.16 ***	0.39 ***	−0.01	0.14 ***
Social Integration	0.40 (0.21)	0.26 ***	−0.11 ***	0.11 ***	0.01

Note: *** *p* < 0.001. PA = positive affect, NA = negative affect, PEMO = positive emodiversity, NEMO = negative emodiversity. SES family education represents the education level of parents and SES family income is family income per past 12 months, with higher values representing greater education and income, respectively.

**Table 2:** Study 1: Positive emodiversity regression analyses.

Outcome Variable, Predictor Variables	*F*(*df*)	*B*	*b*	*t*, *p*	*R* ^2^
Subjective Health	34.14(4,1406) ***				0.09
Positive Affect		5.96	0.28	9.49, <0.001 ***	
PEMO		−1.26	−0.01	0.09, 0.926	
Gender		−3.78	−0.14	5.00, <0.001 **	
Age		−0.35	−0.05	1.95, 0.051	
Satisfaction with Life	193.49(4,1402) ***				0.36
Positive Affect		1.27	0.62	25.51, <0.001 ***	
PEMO		−2.92	−0.07	2.71, 0.007	
Gender		0.07	0.03	1.09, 0.276	
Age		0.01	0.01	0.16, 0.875	
Body Appreciation	59.81(4,1406) ***				0.15
Positive Affect		0.71	0.35	12.55, <0.001 ***	
PEMO		−0.47	−0.01	0.77, 0.439	
Gender		−0.42	−0.16	6.15, <0.001 ***	
Age		0.02	0.03	1.00, 0.319	
Depressive Symptoms	92.39(4,1406) ***				0.21
Positive Affect		−2.22	−0.44	16.41, <0.001 ***	
PEMO		7.66	0.07	2.61, 0.009	
Gender		1.19	0.19	7.33, <0.001 ***	
Age		−0.05	−0.03	1.23, 0.218	
Perceived Stress	133.43(4,1406)				0.28
Positive Affect		−0.58	−0.56	21.74, <0.001 ***	
PEMO		3.08	0.14	5.31, <0.001 **	
Gender		0.18	0.14	5.59, <0.001 **	
Age		0.00	0.00	0.07, 0.942	
Anxiety	134.84(2,1403) ***				0.28
Positive Affect		−0.63	−0.53	20.45, <0.001 ***	
PEMO		3.34	0.13	5.00, <0.001 **	
Gender		0.38	0.25	10.15, <0.001 ***	
Age		0.01	0.04	1.46, 0.145	
Social Integration	34.23(4,1405) ***				0.09
Positive Affect		0.09	0.28	9.52, <0.001 ***	
PEMO		−0.15	−0.02	0.71, 0.478	
Gender		−0.02	−0.04	−1.38, 0.168	
Age		−0.02	−0.16	5.68, <0.001 **	
Loneliness	74.23(4,1406) ***				0.17
Positive Affect		−0.64	−0.44	15.86, <0.001 ***	
PEMO		3.04	0.10	3.47, <0.001 ***	
Gender		0.01	0.01	0.20, 0.839	
Age		−0.04	−0.10	3.82, <0.001 **	

Note: ** *p* < 0.01, *** *p* < 0.001. PEMO = positive emodiversity. For gender, men = 0, women = 1, and age was mean-centered for ease of interpretation. Bonferroni corrections were applied to determine the significance of the regression coefficients, such that significance at the 0.05 level was converted to *p* < 0.006. Due to multicollinearity, several of the directional signs of the beta coefficient values changed for positive emodiversity when positive affect was also included. The magnitude of the main effect of positive affect was larger than positive emodiversity enough to offset the original relationship and switch the direction or sign of that relationship. Proper interpretation of these is made by consulting the original sign of the bivariate correlation (see Table 1).

**Table 3:** Study 1: Negative emodiversity regression analyses.

Outcome Variable, Predictor Variables	*F*(*df*)	*B*	*b*	*t*, *p*	*R* ^2^
Anxiety	392.66(4,1403) ***				0.53
Negative Affect		0.81	0.70	37.04, <0.001 ***	
NEMO		−4.85	−0.28	15.14, <0.001 ***	
Gender		0.14	0.09	4.67, <0.001 ***	
Age		0.01	0.03	1.47, 0.143	
Interpersonal Conflict	64.47(4,1406) ***				0.16
Negative Affect		0.41	0.38	14.92, <0.001 ***	
NEMO		1.00	0.06	2.50, 0.009 *	
Gender		−0.06	−0.05	1.69, 0.091	
Age		−0.01	−0.03	1.21, 0.228	

Note: * *p* < 0.05, *** *p* < 0.001. NEMO = negative emodiversity. For gender, men = 0, women = 1, and age was mean-centered for ease of interpretation. Bonferroni corrections were applied to determine the significance of the regression coefficients, such that significance at the 0.05 level was converted to *p* < 0.025.

**Table 4:** Study 2: Correlations between Time 1 emotional variables and time 2 health variables.

Variable	*M* (*SD*)	T1*α*, T2*α*	T1 PA	T1 NA	T1 PEMO	T1 NEMO
**Demographics**						
Age	23.73 (2.35)	N/A	−0.30 **	−0.01	−0.07	0.06
SES Education	3.48 (1.30)	N/A	−0.13 *	−0.07	0.02	0.01
SES Income	4.20 (1.48)	N/A	0.07	−0.14	0.30 **	−0.01
**Physical Health**						
T2 Subjective Health	74.30 (16.90)	N/A	0.25 **	−0.24 **	0.10	0.01
T2 Physical Symptoms	5.57 (3.75)	N/A	−0.14 *	0.28 **	−0.02	−0.01
**Mental Health**						
T2 Depressive Symptoms	8.93 (3.42)	0.74, 0.79	−0.40 **	0.39 **	−0.22 **	−0.17 **
T2 Satisfaction with Life	4.89 (1.33)	0.83, 0.89	0.52 **	−0.29 **	0.25 **	0.15 *
T2 Body Appreciation	4.97 (1.44)	0.95, 0.97	0.35 **	−0.36 **	0.17 **	0.06
T2 Perceived Stress	2.67 (0.67)	0.82, 0.85	−0.42 **	0.39 **	−0.28 **	−0.11
T2 Anxiety	2.97 (0.77)	0.81, 0.83	−0.36 **	0.45 **	−0.23 **	−0.24 **
**Social Health**						
T2 Loneliness	2.68 (1.04)	0.82, 0.87	−0.35 **	0.33 **	−0.18 **	−0.12
T2 Interpersonal Conflict	2.10 (0.77)	0.84, 0.87	0.18 **	0.33 **	0.00	0.08
T2 Social Integration	0.32 (0.21)	N/A	0.18 **	−0.05	0.02	−0.03

Note: * *p* < 0.05, ** *p* < 0.01. T1 = Time 1 assessment; T2 = Time 2 assessment. PA = positive affect, NA = negative affect, PEMO = positive emodiversity, NEMO = negative emodiversity. Cronbach’s *α* internal consistency estimates are reported for both Time 1 and Time 2 in the third column, as applicable. SES family education represents the education level of parents, and SES family income represents family income per past 12 months, with higher values representing greater education and income, respectively.

**Table 5:** Study 2: Positive emodiversity regression analyses of Time 2 outcomes.

T2 Outcome, T1 Predictor Variables	*F*(*df*)	*B*	*b*	*t*, *p*	*R* ^2^
T2 Satisfaction with Life	41.32(5,229) ***				0.47
T1 Satisfaction with Life		0.64	0.56	8.96, <0.001 ***	
T1 Positive Affect		0.50	0.23	2.99, 0.003 *	
T1 PEMO		−3.76	−0.09	1.52, 0.129	
Gender		0.15	0.06	1.05, 0.295	
Age		0.06	0.09	1.80, 0.073	
T2 Body Appreciation	71.58(5,230) ***				0.61
T1 Body Appreciation		0.73	0.73	16.08, <0.001 ***	
T1 Positive Affect		0.38	0.16	2.86, 0.005 *	
T1 PEMO		−3.94	−0.09	1.72, 0.087	
Gender		−0.02	−0.01	0.13, 0.895	
Age		0.06	0.09	2.00, 0.046	
T2 Depressive Symptoms	29.99(5,230) ***				0.39
T1 Depressive Symptoms		0.55	0.49	7.94, <0.001 ***	
T1 Positive Affect		−1.02	−0.18	2.38, 0.018	
T1 PEMO		2.43	0.02	0.36, 0.723	
Gender		1.03	0.15	2.63, 0.009	
Age		−0.01	−0.01	0.17, 0.863	
T2 Perceived Stress	29.03(5,230) ***				0.39
T1 Perceived Stress		0.52	0.49	7.86, <0.001 ***	
T1 Positive Affect		−0.18	−0.16	2.10, 0.037	
T1 PEMO		−0.01	−0.01	0.01, 0.993	
Gender		0.20	0.15	2.66, 0.008 *	
Age		−0.01	−0.05	0.86, 0.393	
T2 Anxiety	41.98(5,229) ***				0.48
T1 Anxiety		0.66	0.62	11.12, <0.001 ***	
T1 Positive Affect		−0.14	−0.11	1.68, 0.094	
T1 PEMO		0.36	0.02	0.25, 0.801	
Gender		0.04	0.03	0.49, 0.622	
Age		−0.04	−0.11	2.15, 0.032	
T2 Loneliness	43.49(5,230) ***				0.49
T1 Loneliness		0.65	0.62	12.24, <0.001 ***	
T1 Positive Affect		−0.29	−0.17	2.59, <0.010	
T1 PEMO		0.58	0.02	0.30, 0.763	
Gender		0.08	0.04	0.78, 0.434	
Age		−0.04	−0.09	1.71, 0.088	

Note: * *p* < 0.05, *** *p* < 0.001. T1 = Time 1 assessment; T2 = Time 2 assessment. PEMO = positive emodiversity. For gender, men = 0, women = 1, and age was mean-centered for ease of interpretation. Bonferroni corrections were applied to determine the significance of the regression coefficients, such that significance at the 0.05 level was converted to *p* < 0.008.

**Table 6:** Study 2: Negative emodiversity regression analyses of Time 2 outcomes.

T2 Outcome, T1 Predictor Variables	*F*(*df*)	*B*	*b*	*t*, *p*	*R* ^2^
T2 Satisfaction with Life	40.39(5,229) ***				0.47
T1 Satisfaction with Life		0.71	0.63	11.81, <0.001 ***	
T1 Negative Affect		−0.17	−0.08	1.50, 0.136	
T1 NEMO		3.70	0.12	2.42, 0.016 *	
Gender		0.28	0.11	1.96, 0.051	
Age		0.05	0.08	1.49, 0.138	
T2 Depressive Symptoms	29.13(5,229) ***				0.39
T1 Depressive Symptoms		0.58	0.52	7.68, <0.001 ***	
T1 Negative Affect		0.46	0.09	1.24, 0.217	
T1 NEMO		−10.00	−0.13	2.60, 0.010 *	
Gender		0.70	0.10	1.78, 0.077	
Age		0.01	0.01	0.13, 0.893	
T2 Anxiety	42.72(5,229) ***				0.48
T1 Anxiety		0.61	0.57	8.33, <0.001 ***	
T1 Negative Affect		0.14	0.12	1.71, 0.089	
T1 NEMO		−2.16	−0.13	2.53, 0.012 *	
Gender		−0.01	−0.01	0.08, 0.935	
Age		−0.03	−0.10	1.91, 0.057	

Note: * *p* < 0.05, *** *p* < 0.001. T1 = Time 1 assessment; T2 = Time 2 assessment. NEMO = negative emodiversity. For gender, men = 0, women = 1, and age was mean-centered for ease of interpretation. Bonferroni corrections were applied to determine the significance of the regression coefficients, such that significance at the 0.05 level was converted to *p* < 0.017.

**Table 7:** Study 2: Reverse directional regression analyses.

T2 Outcome, T1 Predictor Variables	*F*(*df*)	*B*	*b*	*t*, *p*	*R* ^2^
T2 PEMO	10.86 (2,233) ***				0.09
T1 Positive Affect		0.02	0.33	4.50, <0.001 **	
T1 Depressive Symptoms		0.001	0.09	1.17, 0.242	
T2 PEMO	11.15 (2,232) ***				0.09
T1 Positive Affect		0.02	0.36	4.38, <0.001 **	
T1 Satisfaction with Life		−0.003	−0.12	1.43, 0.153	
T2 PEMO	10.20 (2,233) ***				0.08
T1 Positive Affect		0.02	0.29	4.36, <0.001 **	
T1 Body Appreciation		−0.001	−0.03	0.41, 0.684	
T2 PEMO	10.22 (2,233) ***				0.08
T1 Positive Affect		0.01	0.26	3.48, <0.001 *	
T1 Perceived Stress		−0.002	−0.03	0.45, 0.650	
T2 PEMO	9.98 (2,232) ***				0.08
T1 Positive Affect		0.02	0.28	3.91, <0.001 *	
T1 Anxiety		−0.001	−0.02	0.21, 0.834	
T2 PEMO	10.12 (2,233) ***				0.08
T1 Positive Affect		0.02	0.29	4.29, <0.001 **	
T1 Loneliness		0.00	0.01	0.16, 0.877	
T2 NEMO	6.33 (2,232) **				0.05
T1 Negative Affect		0.01	0.14	1.97, 0.050	
T1 Satisfaction with Life		0.01	0.24	3.50, <0.001 *	
T2 NEMO	9.93 (2,233) ***				0.07
T1 Negative Affect		0.02	0.25	3.10, 0.002 *	
T1 Depressive Symptoms		−0.01	−0.33	4.17, <0.001 **	
T2 NEMO	16.59 (2,232) ***				0.13
T1 Negative Affect		0.02	0.31	4.04, <0.001 **	
T1 Anxiety		−0.03	−0.44	5.72, <0.001 **	

Note: * *p* < 0.05, ** *p* < 0.01, *** *p* < 0.001. T1 = Time 1 assessment; T2 = Time 2 assessment. PEMO = positive emodiversity; NEMO = negative emodiversity. Bonferroni corrections were applied to determine the significance of the regression coefficients, such that significance at the 0.05 level was converted to *p* < 0.005.

## Data Availability

The data that we used for the current study are available from the first author, upon reasonable request.
